# From genes to therapy: navigating the complex landscape of neurofibromatosis management in Canada through advanced diagnostic, targeted therapies, and holistic care

**DOI:** 10.1186/s13023-025-03793-2

**Published:** 2025-08-05

**Authors:** Victor Shu Cheng Zhao

**Affiliations:** Saint George’s School, Vancouver, BC Canada

**Keywords:** Neurofibromatosis, NF-1, NF-2, Schwannomatosis, CRISPR

## Abstract

Neurofibromatosis (NF) presents a significant clinical challenge due to its complex genetic basis, diverse clinical manifestation, and substantial impact on a patient’s quality of life (QoL). This paper explores the multifaceted approach required to manage NF in Canada, emphasizing the integration of advanced diagnostic tools, targeted treatments, and comprehensive support systems. Healthcare providers, researchers, patient advocacy groups, and policymakers must collaborate to ensure NF patients receive the best possible care and support. This disease poses devastating consequences to families, and there has been a lack of awareness of this issue in Vancouver and, generally, in British Columbia. Currently, there is no clinic dedicated explicitly to NF in Metro Vancouver, and patients diagnosed with this disease must be flown to Toronto to get treated. The process is costly and inefficient, demanding changes. Some recent improvements in the field of NF have been noted, such as the use of gene therapy and MEK inhibitors. However, the long-term effect of this treatment is largely unknown and should be viewed with caution. This underscores the importance of enhancing psychological interventions to address the mental health challenges faced by NF patients. Specific gene sequences for different types of NF have also been mentioned in the article to offer insights on potential targets for gene-editing technology like CRISPR. Through ongoing advancements in medical science and a commitment to patient-centered care, this paper envisions significant improvements in the management and treatment of this complex condition.

## Introduction

NF is a rare disease in children that encompasses a group of genetic disorders that cause tumors to form on nerve tissue. The three main types are NF Type 1, NF Type 2, and Schwannomatosis. These conditions can lead to various health issues, including skin changes, bone deformities, and, in some cases, malignancies. NF Type 1 was first characterized in detail by the German pathologist Friedrich von Recklinghausen in 1882. Since then, significant progress has been made in understanding NF’s genetic and molecular basis, though treatment options remain limited [6]. Globally, NF Type 1 occurs in approximately 1 in 3000 individuals, while NF Type 2 is less common, affecting about 1 in 25,000–30,000 people [14]. In Canada, NF is classified as a rare disease, which presents unique challenges in terms of diagnosis, treatment, and support. As such, research specific to the Canadian population is crucial for developing effective healthcare policies and ensuring equitable access to treatment for affected individuals [2]. Recent studies have shown that the QoL for patients with NF in Canada can be significantly impacted, with factors such as pain and physical appearance as major predictors of mental well-being [11]. Moreover, advancements in medical research, such as the development of MEK inhibitors like Selumetinib, which have shown effectiveness in treating plexiform neurofibromas in NF Type 1 patients, are promising [8]. Therefore, a comprehensive understanding of NF in Canadian patients is essential for improving treatment outcomes and developing targeted therapies.

## Types of Neurofibromatosis

### NF1

Neurofibromatosis Type 1 (NF1) arises from mutations in the NF1 gene situated on chromosome 17. This gene encodes neurofibromin, a protein that functions as a tumor suppressor by inhibiting the RAS/MAPK signaling pathway [3]. Mutations in the NF1 gene result in the loss of neurofibromin function, leading to uncontrolled cellular proliferation and tumor development [1]. This loss of regulation underpins the various manifestations of NF1. Clinically, NF1 is characterized by a variety of features, including café-au-lait macules, neurofibromas, Lisch nodules, axillary and inguinal freckling, skeletal abnormalities such as scoliosis and tibial dysplasia, learning disabilities, attention deficit hyperactivity disorder, and an elevated risk for additional tumors, including optic pathway gliomas and malignant peripheral nerve sheath tumors [2, 4, 9, 14, 22]. The diverse symptoms illustrate the complexity of the condition, underscoring the need for a multidisciplinary approach to diagnosis, treatment, and ongoing management.

Globally, the prevalence of NF1 is approximately 1 in 3000 individuals. In Canada, the estimated incidence mirrors this global statistic, ranging from 1 in 2500 to 3000 live births [2, 10]. Research indicates significant gender disparities in QoL among NF1 patients, with women reporting lower scores in physical functioning, emotional well-being, and pain interference compared to men [11]. Furthermore, a cross-sectional study conducted in Toronto revealed that individuals with NF1 had lower employment rates and poorer QoL scores than the general Canadian population [11]. These findings underscore the necessity for targeted interventions to address these QoL disparities and improve the overall well-being of NF1 patients in Canada.

### NF2

Neurofibromatosis Type 2 (NF2) is caused by mutations in the NF2 gene located on chromosome 22. This gene encodes a protein called merlin (or schwannomin), which acts as a tumor suppressor by regulating cell growth and adhesion [20]. Loss of merlin function due to NF2 gene mutations leads to the formation of multiple tumors, particularly in the central nervous system [18]. This disruption in regulation is a critical factor in the pathogenesis of NF2. Standard clinical features of NF2 include bilateral vestibular schwannomas, which lead to hearing loss, tinnitus, and balance issues, as well as meningiomas, ependymomas, early-onset cataracts, and skin tumors [5, 7, 23]. The complexity and severity of these manifestations necessitate a comprehensive approach to management and support for individuals with NF2.

NF2 is less common than NF1, with an estimated prevalence of 1 in 25,000 to 30,000 individuals globally. In Canada, the prevalence is similar, affecting approximately 1 in 30,000 people [14]. Research conducted by the University Health Network in Toronto reported that individuals with NF2 had lower QoL scores in various domains, such as physical functioning and emotional well-being, compared to the general population [12].

High levels of pain and anxiety/depression significantly affect their overall QoL, underscoring the need for comprehensive care and support for those living with NF2.

### Schwannomatosis

Schwannomatosis is caused by mutations in the SMARCB1 or LZTR1 genes, which are involved in regulating cell growth and tumor suppression [20, 21]. Unlike NF1 and NF2, Schwannomatosis primarily affects the peripheral nervous system. This distinction underscores the unique clinical presentation of Schwannomatosis. Standard clinical features of Schwannomatosis include multiple schwannomas, chronic pain, numbness, tingling, or weakness in the affected areas, with less frequent skin manifestations than NF1 and NF2 [13]. These symptoms significantly impact the QoL of individuals with Schwannomatosis.

Schwannomatosis is the rarest form of NF, with a prevalence that is not well established but is believed to be less common than NF1 and NF2 [6, 21]. Data on the prevalence of Schwannomatosis in Canada is limited, necessitating further epidemiological studies to better understand its distribution and impact [16]. Additionally, understanding the prevalence and demographic characteristics of Schwannomatosis can help inform healthcare policy and resource allocation, ensuring that patients receive appropriate care and support.

Enhanced awareness and education about Schwannomatosis among healthcare professionals and the public are also essential for early diagnosis and effective management of the condition.

## Genetic and Molecular Basis

### NF1 Gene and Neurofibromin

The NF1 gene, located on chromosome 17, encodes neurofibromin, a protein that acts as a tumor suppressor by regulating cell growth [3]. Neurofibromin functions as a GTPase-activating protein (GAP) for the RAS protein, converting active RAS-GTP to inactive RAS-GDP, thereby inhibiting the RAS/MAPK signaling pathway involved in cell proliferation and survival [1]. This regulatory role is crucial for maintaining normal cellular functions. The loss or dysfunction of neurofibromin due to NF1 gene mutations leads to hyperactivation of the RAS pathway, resulting in increased cell division and tumor formation [2, 15]. Mutations in the NF1 gene can be inherited or occur spontaneously (de novo), producing a non-functional neurofibromin protein. Consequently, the lack of functional neurofibromin disrupts the regulation of the RAS pathway, leading to uncontrolled cell growth. This disruption results in the development of various tumors, including neurofibromas, optic pathway gliomas, and malignant peripheral nerve sheath tumors.

### NF2 Gene and Merlin

The NF2 gene, located on chromosome 22, encodes merlin (also known as schwannomin), a protein that acts as a tumor suppressor by regulating cell growth and adhesion [20]. Merlin is part of the ezrin-radixin-moesin (ERM) family of proteins and plays a crucial role in linking the actin cytoskeleton to the cell membrane [18]. This structural function is essential for maintaining normal cell morphology and signaling.

Merlin regulates several signaling pathways, including the Hippo, mTOR, and RAS/MAPK pathways, by controlling intercellular communication and inhibiting cell proliferation. The loss of merlin function due to NF2 gene mutations leads to uncontrolled cell growth, resulting in the formation of tumors such as vestibular schwannomas, meningiomas, and ependymomas, predominantly affecting the central nervous system [7]. Such loss of regulation highlights the critical role of merlin in tumor suppression and the maintenance of cellular homeostasis. Understanding the molecular mechanisms underlying NF2 provides insight into potential therapeutic targets and strategies for managing these tumors, emphasizing the importance of ongoing research in this area.

### SMARCB1 and LZTR1 Genes in Schwannomatosis

The SMARCB1 and LZTR1 genes are involved in the regulation of cell growth and tumor suppression [20]. SMARCB1 is part of the SWI/SNF chromatin remodeling complex, which is crucial for gene expression regulation and maintaining genomic stability [13]. This complex plays a vital role in ensuring the proper functioning of cellular processes. LZTR1 is believed to play a role in controlling cell proliferation and apoptosis. Mutations in SMARCB1 or LZTR1 disrupt their normal functions, leading to the formation of multiple schwannomas. These tumors arise from Schwann cells, which form the myelin sheath around peripheral nerves. The mutations inactivate these genes, leading to a loss of function and the Schwann cells proliferating abnormally into schwannomas. Unlike NF1 and NF2, Schwannomatosis primarily affects the peripheral nervous system and is characterized by chronic pain and neurological deficits [6]. This distinction underscores the unique pathophysiology of Schwannomatosis, highlighting the importance of these genes in maintaining normal Schwann cell function and preventing tumor formation.

## Clinical manifestations and diagnosis

### Diagnostic Criteria for NF1

The National Institutes of Health (NIH) has established specific criteria for diagnosing NF1. A diagnosis is made if an individual meets two or more of the following criteria: six or more café- au-lait spots (greater than 5 mm in prepubertal individuals or greater than 15 mm in postpubertal individuals), two or more neurofibromas of any type or one plexiform neurofibroma, freckling in the axillary or inguinal regions, optic glioma, two or more Lisch nodules (iris hamartomas), a distinctive osseous lesion such as sphenoid dysplasia or tibial pseudoarthrosis, or a first-degree relative with NF1 based on the above criteria [3]. It is also important to note that recent research breakthroughs have found a correlation between choroidal abnormalities (CA) and NF1. Due to the high specificity of this condition in relation to NF1, CA has been added as an ocular diagnostic criterion of NF1 as an alternative to Lisch nodules [19]. This set of criteria ensures a comprehensive evaluation for accurate diagnosis.

Common signs and symptoms of NF1 include skin manifestations like café-au-lait spots, cutaneous neurofibromas, and freckling. Neurological manifestations include learning disabilities, attention deficit hyperactivity disorder (ADHD), seizures, and headaches.

Ophthalmological issues can manifest as Lisch nodules and optic gliomas.

Additionally, skeletal abnormalities like scoliosis, tibial dysplasia, and other bone deformities are prevalent. Individuals with NF1 have an increased risk of tumors, including malignant peripheral nerve sheath tumors and different types of cancer [1, 2].

These diverse manifestations underscore the complexity and multi-system involvement of NF1. Diagnosis of NF1 often involves a combination of clinical evaluation, imaging studies, and genetic testing. Magnetic resonance imaging (MRI) is commonly used to identify internal neurofibromas and optic gliomas. Genetic testing can confirm mutations in the NF1 gene, providing a definitive diagnosis [2]. This allows for accurately identifying and managing the condition, facilitating targeted intervention, and ongoing monitoring for associated complications.

### Diagnostic Criteria for NF2

The Manchester diagnostic criteria for NF2 include bilateral vestibular schwannomas confirmed by MRI or a first-degree relative with NF2, unilateral vestibular schwannoma, or any two of the following: meningioma, schwannoma, glioma, neurofibroma, or posterior subcapsular lenticular opacities [18]. This comprehensive criteria set ensures a thorough and accurate diagnosis.

Common signs and symptoms of NF2 include hearing loss, tinnitus, and balance problems due to vestibular schwannomas. Meningiomas can cause headaches, seizures, and other neurological deficits. Spinal tumors may lead to pain, weakness, and sensory changes. Eye issues such as cataracts can occur early, and skin tumors and café-au-lait spots are less common than in NF1 [20]. These symptoms highlight the multi-faceted nature of NF2 and its impact on various bodily systems. MRI is the primary imaging modality for detecting vestibular schwannomas, meningiomas, and spinal tumors. Genetic testing can identify mutations in the NF2 gene, confirming the diagnosis.

### Diagnostic Criteria for Schwannomatosis

The diagnosis of Schwannomatosis is based on the presence of multiple schwannomas without vestibular schwannomas and the lack of a clear family history of NF2. Genetic testing can identify mutations in the SMARCB1 or LZTR1 genes [20]. The diagnostic criteria include two or more non-intradermal schwannomas confirmed by histology, no evidence of vestibular schwannomas on high-quality MRI with thin slices through the internal auditory canals, and genetic testing excluding mutations in the NF2 gene [13]. These criteria ensure a precise and thorough diagnosis of Schwannomatosis. Typically, Schwannomatosis symptoms include multiple schwannomas causing chronic pain and neurological deficits. Symptoms such as numbness, tingling, or weakness in affected areas are also prevalent. Skin manifestations are less frequent compared to NF1 and NF2. MRI is used to identify schwannomas and rule out vestibular schwannomas, while genetic testing focuses on identifying mutations in the SMARCB1 and LZTR1 genes. Specialized centers in Canada provide the necessary diagnostic tools and expertise to diagnose Schwannomatosis accurately [6]. These centers are crucial in managing the condition, offering advanced imaging and genetic testing capabilities that facilitate comprehensive care for affected individuals.

## Management and Treatment in Canada

### Medical Management

Pharmacological management of NF in Canada includes the use of pain relievers, anticonvulsants, and targeted therapies. Pain management is crucial for patients with painful neurofibromas and schwannomas. Anticonvulsants are used to manage seizures associated with NF1 and NF2. Targeted therapies, such as MEK inhibitors (e.g., Selumetinib), have shown promising results in treating plexiform neurofibromas in NF1 patients [8]. These treatments are essential for improving QoL and managing the complex symptoms of NF. New research and clinical trials are continuously being conducted to find more effective treatments and enhance existing therapies for the illness. Access to advanced medical care and ongoing support systems is vital for ensuring patients receive the most current and effective treatment options.

Chronic pain is a significant issue for many NF patients, particularly those with Schwannomatosis. Pain management strategies include the use of nonsteroidal anti-inflammatory drugs (NSAIDs), opioids for severe pain, and adjuvant therapies such as antidepressants and anticonvulsants. Multidisciplinary pain clinics offer comprehensive pain management plans, including physical therapy and psychological support [3]. This holistic approach is crucial for addressing the multifaceted nature of chronic pain in NF patients.

Effective management of NF requires ongoing surveillance care to monitor disease progression, detect complications early, and provide timely interventions. Surveillance is particularly critical in pediatric patients, as early intervention can significantly improve outcomes.

During infancy and early childhood, annual dermatological and neurological evaluations are crucial for detecting early signs of NF, such as café-au-lait macules and neurofibroma development. Ophthalmological screening is recommended by 12 months of age to check for optic pathway gliomas (OPGs), followed by annual evaluations until age eight [2]. Additionally, developmental assessments should be conducted to monitor potential cognitive and motor delays. As children grow older, between the ages of 6 and 12, neurological and musculoskeletal evaluations every six to twelve months are necessary to check for scoliosis and tibial dysplasia, while ophthalmological evaluations should continue every one to two years if no previous signs of OPGs are present. Cardiac screening may be required for patients with suspected vascular abnormalities [20].

For adolescents aged 13–18, annual assessments focus on monitoring neurofibroma growth, particularly for plexiform neurofibromas, while hearing evaluations help detect NF2-related vestibular schwannomas. MRI scans become increasingly crucial if symptoms suggest spinal tumors or progressive neurological deficits. In adulthood, routine clinical evaluations every one to two years provide continued tumor monitoring and pain management. For NF2 patients, MRI scans are recommended every two to three years to track tumor progression. Additionally, genetic counseling plays a key role in family planning and risk assessment [18].

Surveillance care in NF management requires coordination among various specialists. Neurologists assess for headaches, cognitive deficits, and tumor growth, while ophthalmologists focus on detecting and managing optic gliomas and cataracts associated with NF1 and NF2.

Orthopedic specialists monitor scoliosis and long bone dysplasia, while oncologists oversee plexiform neurofibromas and malignant peripheral nerve sheath tumors (MNPSTs). Genetic counselors provide risk assessments and family planning advice, and psychologists offer mental health support to address anxiety and depression in NF patients [11]. Ensuring comprehensive surveillance through a structured and collaborative approach improves patient outcomes and enhances the QoL for individuals living with NF.

Genetic counseling is also integral to managing NF, helping patients understand their condition and the risks of inheritance to offspring [1]. These ongoing assessments and interventions ensure proactive management of the disease and its complications. In addition to medical and genetic evaluations, psychosocial support is essential for addressing the emotional and mental health challenges faced by NF patients and their families. Collaboration between healthcare providers, including neurologists, geneticists, oncologists, and mental health professionals, is essential for providing comprehensive care and improving patient outcomes.

### Surgical Management

Surgery is often indicated for symptomatic neurofibromas, schwannomas, and other tumors that cause significant pain, neurological deficits, or have a risk of malignant transformation. In NF1, surgical resection of plexiform neurofibromas may be necessary to alleviate pain or prevent complications from tumor growth. For NF2, surgical removal of vestibular schwannomas can help preserve hearing and prevent further neurological damage [6]. Surgical techniques include microsurgery, endoscopic surgery, and stereotactic radiosurgery. The choice of technique depends on the tumor location, size, and associated risks. However, the outcomes of these surgeries vary, intending to maximize tumor removal while minimizing neurological damage.

Complications can include infection, bleeding, and nerve damage, making surgical expertise critical [7]. The risks associated with surgery in NF patients include incomplete tumor removal, recurrence, and post-operative complications. Access to specialized surgical centers and experienced neurosurgeons in Canada improves the management of these risks.

Multidisciplinary teams, including neurosurgeons, oncologists, and rehabilitation specialists, ensure comprehensive care and better outcomes for NF patients [18]. These experts work collaboratively to develop individualized treatment plans, addressing the immediate surgical needs and the long-term care required for optimal patient health and QoL.

### Emerging Therapies

One of the most promising developments in the field is targeted therapies, which have shown significant promise in managing NF. In Canada, MEK inhibitors like selumetinib have been approved for treating symptomatic and inoperable plexiform neurofibromas in pediatric NF1 patients. Clinical trials have demonstrated tumor shrinkage and improvement in related morbidities with Selumetinib treatment [8]. This approval marks a significant advancement in the therapeutic options available for NF1 patients as it provides a non-surgical treatment alternative. Also, advances in gene therapy offer potential future treatment options for NF. Research is focused on correcting the underlying genetic mutations in NF1, NF2, and Schwannomatosis. These therapies aim to restore normal gene function and prevent tumor formation. Although still in experimental stages, gene therapy represents a promising avenue for long-term management and potential cure of NF [6]. The ability to directly address genetic mutation using technology such as CRISPR could revolutionize NF treatment, providing hope for more effective and lasting solutions.

Furthermore, numerous clinical trials are ongoing in Canada, exploring various targeted therapies, gene therapies, and novel drug combinations. These trials are essential for developing new treatments and improving existing ones. Collaboration between Canadian research institutions, healthcare providers, and patient advocacy groups is vital for advancing NF research and ensuring patients have access to cutting-edge treatments [7]. Working together allows these groups to pool resources, share knowledge, and accelerate the development of effective therapies. Additionally, such collaborations help raise awareness and secure funding for NF research, which is essential for sustaining progress in this field.

## Healthcare System and Policy Implications

### Rare Disease Framework in Canada

Canada has established policies and frameworks to support individuals with rare diseases, including NF. The Canadian Organization for Rare Disorders (CORD) plays a pivotal role in advocating for policy changes and implementing national strategies for rare disease management. The Canadian Rare Disease Strategy outlines goals for improving diagnosis, treatment, and access to care for rare disease patients (Canadian Cancer Society). Access to specialized care for NF patients is facilitated through dedicated clinics and centers of excellence, such as the Neurofibromatosis Clinic Network. These centers provide comprehensive care, including genetic counseling, regular monitoring, surgical interventions, and access to clinical trials.

However, there are disparities in access to care based on geographic location, emphasizing the need for equitable healthcare distribution [8]. Both public and private sectors support funding for rare disease research and treatment in Canada. Government funding through agencies like the Canadian Institutes of Health Research (CIHR) and initiatives like the Rare Disease Foundation provide essential resources for advancing NF research and improving patient care. Additionally, patient advocacy groups and non-profit organizations contribute to raising awareness and securing funding for rare diseases (CSPA—Neurofibromatosis). Integrating these efforts into the broader healthcare system is essential for sustaining progress. Continuous support and policy development are needed to address the evolving challenges of rare disease management, ensuring that all patients receive the care and attention they deserve.

### Challenges and Barriers

One of the significant challenges in managing NF is the delay in diagnosis. Symptoms of NF can vary widely and often overlap with other conditions, leading to misdiagnosis or late diagnosis.

Early genetic testing and increased awareness among healthcare providers are crucial to reduce these delays and improve patient outcomes (New Neurofibromatosis Type 1 Findings, 2020) [12]. Despite advancements in targeted therapies and surgical techniques, access to these treatments can be limited, particularly for patients in remote or underserved areas. The high costs of novel treatments and the need for specialized care centers pose additional barriers. Policies to subsidize costs and improve the distribution of specialized care are needed to ensure all patients receive timely and effective treatment [2].

Geographic location and socioeconomic status significantly impact access to care and treatment outcomes for NF patients in Canada. Rural and remote areas often lack specialized healthcare facilities, leading to disparities in the quality of care received. Socioeconomic factors, including income and insurance coverage, also affect patients’ ability to access necessary treatments and participate in clinical trials [14]. Addressing these disparities requires targeted policy interventions to improve healthcare infrastructure in underserved areas and financial support mechanisms to alleviate the burden on patients.

Furthermore, continuous education and training for healthcare professionals are essential to recognize and manage NF effectively. Multidisciplinary approaches involving specialists, such as neurologists, oncologists, and genetic counselors, are vital for comprehensive patient care. Enhancing collaboration between healthcare providers, policymakers, and patient advocacy groups can lead to more equitable healthcare distribution and better outcomes for NF patients across Canada.

### Support Systems

Addressing the challenges and barriers in managing NF requires a multifaceted approach in which patient advocacy groups and support networks are indispensable. Patient advocacy groups like the Canadian Neurofibromatosis Foundation (CNFF) and other rare disease organizations are crucial in supporting NF patients. They provide resources, raise awareness, advocate for policy changes, and fund research initiatives. These groups also offer support networks for patients and families, helping them navigate the healthcare system and access necessary services (Canadian Cancer Society).

Living with NF can have significant psychological impacts, including anxiety, depression, and social isolation [11]. Psychological support and counseling are essential components of comprehensive care for NF patients. Multidisciplinary teams that include mental health professionals can provide tailored support to address these challenges and improve overall QoL [17]. Integrating mental health services into the care plan ensures that patients’ emotional and psychological needs are met alongside their physical health requirements.

Community-based resources and support networks provide additional assistance to NF patients and their families. These may include local support groups, educational workshops, and online forums where individuals can share experiences and advice. Access to these resources helps build a sense of community and provides valuable emotional and practical support [13]. They help alleviate feelings of isolation and empower individuals to manage their condition more effectively by fostering connections among patients and families. Overall, the involvement of advocacy groups and community resources enhances the support system for NF patients, contributing to better health outcomes and an improved QoL.

## Research and Future Directions

### Current Research Trends in Canada

Canada is at the forefront of NF research, with numerous clinical trials underway that explore a variety of innovative treatment approaches. These trials include investigations into the efficacy of MEK inhibitors, promising gene therapy techniques, and novel combination therapies. For instance, the University Health Network in Toronto spearheads several groundbreaking studies to enhance the understanding and treatment of NF1 and NF2 [8]. Such initiatives are crucial in driving the therapeutic landscape forward, offering patients renewed hope.

In addition to clinical trials, Canadian research efforts are delving into epidemiological studies to better understand the prevalence, incidence, and natural history of NF within the population.

Research conducted by Barnett et al. [2] and Lee et al. [14] yields invaluable data that inform healthcare policies and optimize resource allocation for NF patients. This information is instrumental in shaping public health strategies and ensuring that resources are deployed where they are most needed.

QoL research is another critical area of focus, aiming to develop comprehensive treatment plans that address the multifaceted impact of NF. Recent studies have highlighted notable gender disparities in QoL, with women reporting lower scores in physical functioning, emotional well- being, and pain management. Insights from these studies, such as those by Hamoy-Jimenez et al. [11], are essential for creating tailored support and intervention strategies that enhance the overall well-being of NF patients. These findings emphasize the need for personalized care approaches that consider the diverse experiences of different patient groups.

The collaborative research environment in Canada, supported by institutions, healthcare providers, and patient advocacy groups, is fostering significant advancements in the field of NF. Continued investment in research and the seamless integration of new discoveries into clinical practice is vital for achieving better patient outcomes and improving the QoL for those affected by NF. Through these concerted efforts, Canada is making substantial progress in understanding and treating this complex condition.

### Future Research Area

The future of NF research is brimming with potential, yet several critical areas demand more in- depth exploration to unlock innovative treatments and improve patient outcomes. While the introduction of therapies like MEK inhibitors and gene therapy has been groundbreaking, their long-term efficacy and safety remain largely uncharted. Conducting extensive longitudinal studies will validate these treatments and help to integrate them into the healthcare system for maximum benefit and minimal risk [1]. Personalized medicine is on the cusp of transforming NF treatment. By leveraging genetic profiling and biomarker analysis, researchers can identify which patients will respond best to specific therapies, paving the way for highly targeted and effective treatments [6].

Advanced diagnostic tools represent a thrilling frontier in the quest for earlier and more accurate NF diagnosis. Cutting-edge imaging technologies and sophisticated genetic tests are poised to enhance our ability to detect NF early and monitor its progression with unparalleled precision [14]. Addressing the psychological and social challenges faced by NF patients is equally crucial. Innovative psychosocial interventions, including robust support systems, tailored counseling programs, and vibrant community resources, are essential to improving the QoL for those living with NF [11]. Furthermore, exploring novel therapeutic avenues and diving deep into the molecular underpinnings of NF can unveil unprecedented treatment possibilities. Collaborative efforts between researchers, clinicians, and patient advocacy groups are vital to accelerating these discoveries and translating them swiftly into clinical practice. However, despite these promising developments, there remains a pressing need for increased research and investment in this field to realize the full potential of these innovations. This combination of expertise and passion drives the next wave of NF breakthroughs, promising a brighter, more balanced future for NF patients.

## Conclusion

NF embodies a profound clinical challenge rooted in its intricate genetic foundation and diverse manifestations. In Canada, managing NF as a rare disease, particularly affecting children, necessitates a sophisticated approach, integrating advanced diagnostics, targeted therapies, and comprehensive support systems. While the healthcare framework facilitates specialized care and access to innovative treatments, hurdles like diagnostic delays, limited treatment accessibility, and socioeconomic disparities persist. The advent of targeted therapies like MEK inhibitors opens a new era of promising outcomes, and the potential of gene therapy looms on the horizon. Long-term studies, personalized medicine, and enhanced diagnostic tools are essential for future advancements. Moreover, bolstering psychosocial support is critical to improving the holistic well-being of NF patients. Through the concerted efforts of healthcare providers, researchers, patient advocates, and policymakers, there lies many hopes for transformative progress in treating and managing NF in Canada.

## Data Availability

The datasets used and/or analyzed during the current study are available from the corresponding author upon reasonable request.
